# New insights into the key role of HIF-1α in IL-10-producing B cells

**DOI:** 10.15698/cst2018.04.133

**Published:** 2018-03-22

**Authors:** Xianyi Meng, Aline Bozec

**Affiliations:** 1 Department of Internal Medicine 3, Friedrich-Alexander-University Erlangen-Nürnberg (FAU) and Universitätsklinikum Erlangen, Erlangen, 91054, Germany.

**Keywords:** HIF-1α, IL-10, B cells, glycolysis, metabolism, autoimmune disease

## Abstract

Hypoxia-inducible factors (HIFs) are essential transcription factors for the cellular response to hypoxia. Expression and stabilization of HIFs can be triggered by hypoxia or by other factors under pathological stress such as inflammation and infection. Indeed, regulatory function of HIFs has been implicated in a variety of different immune cells like macrophages and T cells. In our recent study, we delineated HIF-1α and HIF-2α roles in B cells (Nat Commun, 9:251). We demonstrated that lack of HIF-1α in B cells leads to impaired IL-10-producing CD1d^hi^CD5^+^ B cells expansion by modulating their glycolytic metabolism. We identified HIF-1α as a critical transcriptional factor involved in IL-10 production by B cells, thereby influencing the course of autoimmune diseases.

HIFs are heterodimeric transcription factors, consisting of an oxygen-labile alpha subunit (HIF- α) and a constitutively-stable beta subunit (HIF-β), that exert pivotal roles in inducing cellular responses to hypoxia. HIF-1α and HIF-2α are hydroxylated by prolyl hydroxylases (PHD) and degraded after binding to protein von Hippel Lindau (pVHL) under normoxic conditions. Our *in vitro *analyses demonstrated that LPS or BCR-mediated activation of B cells can also induce the expression of HIF-1α, but not HIF-2α, in an oxygen-independent way. HIF-1α expression in BCR-stimulated B cells is promoted by the ERK-STAT3 signaling pathway. Following B cell activation, phosphorylated STAT3 at position Ser727 transcriptionally induces the *Hif1*α gene.

We also explored the role of HIFs during B lymphocytes development *in vivo* by crossing mice carrying a loxP-flanked *Hif1*α or *Hif2*α allele with mice expressing cre recombinase from the *Mb1* promoter (referred to herein as *Mb1^cre^Hif1*α*^f/f ^*or *Mb1^cre^Hif2*α*^f/f ^*mice). Our results showed that HIF-2α has no essential role during B cell development, whereas HIF-1α is important for B1a population in the peritoneum. B1a cells possess regulatory functions and produce the anti-inflammatory cytokine IL-10 after activation. Next, we focused on whether IL-10 is altered by loss of HIF-1α in B cells. Indeed, we observed that the frequency of IL-10-producing B cells in bone marrow, spleen, inguinal lymph nodes and the peritoneal cavity were decreased in *Mb1^cre^Hif1*α*^f/f ^*mice. Our findings showed that HIF-1α is an important factor for IL-10-producing B cells.

HIF-1α has previously been shown to act as a key factor in the reprogramming of immune cells metabolism by activating transcription of genes encoding glucose transporters and glycolytic enzymes, which import glucose and convert it into lactate. HIF-1α-mediated shift from oxidative to glycolytic metabolism regulates immune cell function and proliferation. We found that CD1d^hi^CD5^+^ B cells displayed a 2-fold increase in glucose transport activity compared to CD1d^lo^CD5^-^ B cells, suggesting that CD1d^hi^CD5^+^ B cells preferentially use glucose metabolism. Moreover, CD1d^hi^CD5^+^ B cells from *Mb1^cre^Hif1*α*^f/f ^*mice exhibited a lower level of glucose uptake and lactate secretion compared to CD1d^hi^CD5^+^ B cells from WT mice. Accordingly, mRNA expression of HIF-1α -targeted glycolytic enzymes, *Glut1*, *Pkm2*, *Hk2*, *Ldha*, *Pdk1* and *Gpi1*, were markedly decreased in *Hif1*α-deficient compared to WT CD1d^hi^CD5^+^ B cells. Our study showed for the first time that glycolytic metabolism of CD1d^hi^CD5^+^ B cells depends on HIF-1α expression and that HIF-1α contributes to CD1d^hi^CD5^+^ B cell expansion. This work provided a new insight into the role of HIF-1α for IL-10-producing B cells.

Next, we delineated the physiological relevance of the immunoregulation of HIF-1α on IL-10-producing B cells and the protective role of HIF-1α-deficient B cells in autoimmune disease. Our data showed that impaired IL-10 production by HIF-1α-dependent B cells is associated with decreased Tr1 cells, indicating a regulatory network between IL-10-producing B cells and Tr1 cells. In addition, increased IL-17 and IFN-γ production in *Hif1*α-deficient mice is associated with a strong exacerbation of experimental autoimmune encephalomyelitis (EAE) and inflammatory arthritis. Our findings indicate that HIF-1α is a key element for IL-10-producing B cell proliferation and IL-10 signaling, thereby influencing the course of T cell mediated autoimmune diseases such as EAE and arthritis (**Figure 1**).

**Figure 1:**
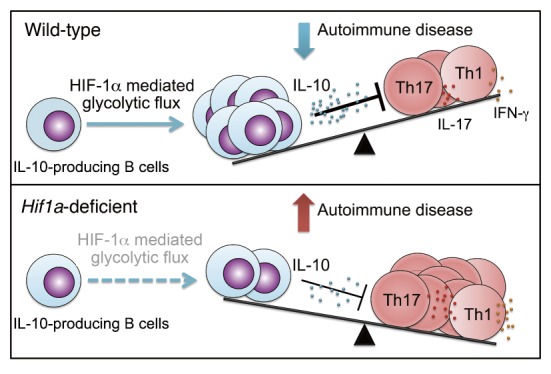
FIGURE 1: Role of HIF-1α-mediated glycolysis in expansion and function of IL-10-producing B cells.

In summary, we provide a novel molecular mechanism for the regulation of IL-10 producing B cells by HIF-1α. By modulating glycolytic metabolism, HIF-1α regulates IL-10-producing B cell expansion. Moreover, we identified HIF-1α as a critical node involved in IL-10 production by B cells. HIF-1α effectively binds to hypoxia response elements in the *Il10 *promoter, leading to induced expression of B cell derived IL-10. In consequence, HIF-1α expression in B cells regulates autoimmune disease.

One of the most obvious unanswered questions is whether a glycolytic gene signature controlling expansion of IL-10-producing B cells exist. Data from our group show that many glycolytic genes such as *Glut1*, *Pkm2*, *Hk2*, *Ldha*, *Pdk1* and *Gpi1*, were decreased in *Hif1*α-deficient CD1d^hi^CD5^+^ B cells. B lymphocytes must adapt to a wide array of environmental stressors as part of their normal development, where they undergo a dramatic metabolic remodelling processes. Recent studies, including ours, provided important proof that metabolic remodeling in B cells during their differentiation or proliferation is essential for their functions. Future studies might provide new proof of principle for the role of energy-conversion pathway in B cell differentiation.

The function of HIFs in B cells is of critical importance not only for fundamental knowledge, but also for further therapeutic opportunity. Indeed, it is possible to target the functions of HIFs using small PHD inhibitors. It is however essential to further understand the possible off-target effects of these drugs during diseases treatment such as for intestinal disorders, arthritis and tumorigenesis.

